# Supplementary material for: Coverage and determinants of deworming uptake among under-five children in Somalia: A multilevel analysis of the 2020 SDHS data
Source: PLoS One. 2025 Nov 14;20(11):e0336429. doi: 10.1371/journal.pone.0336429 (PMC12617879; doi:10.1371/journal.pone.0336429)
Supplement: S1 Table — (DOCX) [file pone.0336429.s001.docx]

**Table S1:** Multivariable multilevel analysis result of poor deworming among children of 12–59 months in Somalia

| **Variables** | **Categories** | **Model I**  **(Null model )** | **Model II**  **AOR (95% CI)** | **Model III**  **AOR (95% CI )** | **Model IV**  **AOR (95% CI**) |
| --- | --- | --- | --- | --- | --- |
| Maternal age | 15-24 |  | Ref |  | Ref |
|  | 25-34 |  | 0.96(0.80,1.14) |  | 0.89(0.75,1.07) |
|  | 35-49 |  | **0.78(0.63,0.97)*** |  | **0.73(0.59,0.90)*** |
| Maternal education | No education |  | Ref |  | Ref |
|  | Primary |  | **0.72(0.59,0.87)*** |  | **0.69(0.56,0.84)*** |
|  | Secondary |  | 0.87(0.60,1.25) |  | 0.84(0.58,1.22) |
|  | Higher |  | **0.32(0.19,0.55)*** |  | **0.35(0.20,0.60)*** |
| Family Wealth status | Poor |  | Ref |  | Ref |
|  | Middle |  | **0.33(0.26,0.40)*** |  | **0.39(0.32,0.49)*** |
|  | Rich |  | **0.31(0.25,0.38)*** |  | **0.36(0.29,0.45)*** |
| Health-related decision-making autonomy | Mother alone |  | Ref |  | Ref |
|  | Husband alone |  | **0.67(0.54,0.82)*** |  | **0.74(0.60,0.91)*** |
|  | Jointly |  | **0.75(0.60,0.93)*** |  | **0.76(0.61,0.95)*** |
| Working status | Nor working |  | Ref |  | Ref |
|  | Working |  | 0.26(0.15,0.45) |  | **0.28(0.16,0.48)*** |
| Perceived distance to health facilities | Not a big problem |  | Ref |  | Ref |
|  | Big problem |  | 0.98(0.84,1.15) |  | 0.99(0.84,1.17) |
| Place of delivery | Home |  | Ref |  | Ref |
|  | Health facility |  | **0.63(0.53,0.74)*** |  | **0.59(0.49,0.70)*** |
| Sex of household head | Male |  | Ref |  | Ref |
|  | Female |  | 1.11(0.95,1.31) |  | 1.07(0.91,1.26) |
| Sex of the child | Male |  | Ref |  | Ref |
|  | Female |  | 0.96(0.83,1.11) |  | 0.97(0.84,1.12) |
| Age of the child (months) | 12–15 |  | Ref |  | Ref |
|  | 16–19 |  | 0.79(0.54,1.17) |  | 0.77(0.51,1.14) |
|  | 20–59 |  | **0.76(0.60,0.97)*** |  | **0.77(0.60,0.98)*** |
| Had diarrhea recently | Yes |  | Ref |  | Ref |
|  | No |  | **6.28(5.16,7.65)*** |  | **6.26(5.11,7.67)*** |
| Place of residence | Rural |  |  | Ref | Ref |
|  | Urban |  |  | **0.72(0.60,0.87)*** | **0.65(0.51,0.82)*** |
|  | Nomadic |  |  | **0.40(0.34,0.48)*** | **0.40(0.32,0.49)*** |
| Region | Awdal |  |  | Ref | Ref |
|  | Woqooyi Galbeed |  |  | 0.40(0.34,0.48) | 0.68(0.40,1.14) |
|  | Togdheer |  |  | **0.31(0.21, 0.47)*** | **0.43(0.27,0.70)*** |
|  | Sool |  |  | 0.46(0.31,0.69) | 0.57(0.35,0.93) |
|  | Sanaag |  |  | **0.41(0.27,0.61)*** | **0.47(0.29,0.75)*** |
|  | Bari |  |  | **0.43(0.28,0.65)*** | **0.47(0.28,0.79)*** |
|  | Nugaal |  |  | **0.64(0.41,1.00)*** | **0.51(0.30,0.85)*** |
|  | Mudug |  |  | 0.74(0.47,1.17) | 0.56(0.33,0.96) |
|  | Galgaduud |  |  | 0.91(0.57,1.48) | 0.76(0.43,1.33) |
|  | Hiraan |  |  | 1.17(0.71,1.92) | 1.16(0.63,2.14) |
|  | Middle Shabelle |  |  | 0.79(0.49,1.25) | 0.94(0.54,1.65) |
|  | Banadir |  |  | **0.56(0.37,0.85)*** | **0.62(0.38,1.01)*** |
|  | Bay |  |  | 0.57(0.32,1.00) | 0.57(0.30,1.07) |
|  | Bakool |  |  | 0.74(0.47,1.15) | 0.69(0.42,1.16) |
|  | Gedo |  |  | 1.13(0.70,1.82) | 0.79(0.45,1.40) |
|  | Lower Juba |  |  | **0.29(0.19,0.44)*** | **0.33(0.20,0.53)*** |
| **AIC** | | 8494.361 | 5554.869 | 8190.736 | 5424.442 |
| **BIC** | | 8501.982 | 5685.846 | 8327.909 | 5679.119 |
| **Likelihood** | | -4246.181 | -2759.434 | -4077.368 | -2677.221 |

**Note:** *: $p$<0.05; AIC: Akaike Information Criterion; BIC: Bayesian Information Criterion
