# Supplementary material for: Coverage and determinants of deworming uptake among under-five children in Somalia: A multilevel analysis of the 2020 SDHS data
Source: PLoS One. 2025 Nov 14;20(11):e0336429. doi: 10.1371/journal.pone.0336429 (PMC12617879; doi:10.1371/journal.pone.0336429)
Supplement: Attachment — Submitted filename: Point-by-point response to reviewers.docx [file pone.0336429.s004.docx]

**Authors' responses to the review comments:**

We would like to sincerely thank the anonymous reviewers and the Academic Editor for their valuable comments. We have considered all comments and then thoroughly revised and formatted the manuscript. A detailed response to each comment is provided in the tables as follows.

| **Editor comments:** | **Response** | **Note** |
| --- | --- | --- |
| Thank you for submitting your manuscript to PLOS ONE. After careful consideration, we feel that it has merit but does not fully meet PLOS ONE’s publication criteria as it currently stands. Therefore, we invite you to submit a revised version of the manuscript that addresses the points raised during the review process.  Please submit your revised manuscript by Nov 29 2025 11:59PM. If you will need more time than this to complete your revisions, please reply to this message or contact the journal office at plosone@plos.org. When you're ready to submit your revision, log on to https://www.editorialmanager.com/pone/ and select the 'Submissions Needing Revision' folder to locate your manuscript file.  Please include the following items when submitting your revised manuscript:  A rebuttal letter that responds to each point raised by the academic editor and reviewer(s). You should upload this letter as a separate file labeled 'Response to Reviewers'.  A marked-up copy of your manuscript that highlights changes made to the original version. You should upload this as a separate file labeled 'Revised Manuscript with Track Changes'.  An unmarked version of your revised paper without tracked changes. You should upload this as a separate file labeled 'Manuscript'.  If you would like to make changes to your financial disclosure, please include your updated statement in your cover letter. Guidelines for resubmitting your figure files are available below the reviewer comments at the end of this letter. | Thank you very much for your comments and feedback. We believe that it helps to enhance the quality of the manuscript. As per comments, a careful revision has been conducted, and all required files are uploaded to the journal submission system. | The revised texts are highlighted in “red” color. |
| **Journal Requirements:** | **Response** | **Note** |
| 1. If the reviewer comments include a recommendation to cite specific previously published works, please review and evaluate these publications to determine whether they are relevant and should be cited. There is no requirement to cite these works unless the editor has indicated otherwise. | Thank you very much. We checked it carefully. |  |
| 2. Please review your reference list to ensure that it is complete and correct. If you have cited papers that have been retracted, please include the rationale for doing so in the manuscript text, or remove these references and replace them with relevant current references. Any changes to the reference list should be mentioned in the rebuttal letter that accompanies your revised manuscript. If you need to cite a retracted article, indicate the article’s retracted status in the References list and also include a citation and full reference for the retraction notice. | Thanks. We checked the reference list and all are OK. |  |
| **Reviewer 1 Comments:** | **Response** | **Note** |
| Dear editor, thank you for the opportunity to review the revised version of the manuscript titled "Coverage and Determinants of Deworming Uptake among Under-Five Children in Somalia: A Multilevel Analysis of the 2020 SDHS Data"  Manuscript ID: PONE-D-25-36783  I have carefully evaluated the authors' responses to my initial comments and appreciate their efforts in addressing most of the concerns raised. However, two important issues remain unresolved:  1. Age Range for Deworming Coverage: The authors continue to report deworming coverage among children aged 12–59 months. However, children aged 12–23 months are not typically included in the target population for deworming in many national programs, where the standard target group is children aged from 24 months. I recommend that the authors either:   Re-analyze or reframe their findings using the 24–59 month age group, in line with national and international deworming guidelines, after confirming the age-specific data in the 2020 SDHS, or   Provide a clear justification—supported by relevant national or international policy or published literature—for including children aged 12–23 months in the analysis to make for readers clear. | Thank you very much for your comments and feedback. We believe that it helps to enhance the quality of the manuscript. We revised the manuscript as per your review comments.  We checked and revised our manuscript based on the published literature on children aged 12-59 months. The following. Some of them are given below.  <https://doi.org/10.1371/journal.pone.0297377>  <https://doi.org/10.1371/journal.pntd.0006500>  <https://doi.org/10.1016/j.jegh.2013.12.005>  <https://doi.org/10.1155/2023/9529600>  <https://dhsprogram.com/Data/Guide-to-DHS-Statistics/Micronutrient_Supplementation_and_Deworming_among_Children.htm> | Revised texts are in “red”. Page: 4 |
| 2. Inconsistent Confidence Interval Reporting: In the statement “The overall prevalence of poor deworming uptake in Somalia was 92%”, the previously cited 95% confidence interval (10.67–11.99) does not correspond to the reported point estimate and appears to be an error. This discrepancy has not been addressed in the revision. The authors should correct this by reporting an accurate and appropriate confidence interval consistent with the prevalence estimate.  Recommendation: Minor Revision  With these remaining issues adequately addressed, the manuscript would be suitable for publication. | Thanks for your suggestion. We appreciate it. It was a typo, so we removed it from the manuscript. As per your comments, we have added the corrected 95%CI in the manuscript. | Revised texts are in “red”. Page: 6 |
| **Reviewer 2 Comments:** | **Response** | **Note** |
| The authors have addressed all the comments raised by the reviewers. The authors may consider to use a forest plot instead of table for presenting the results of the regression models.  Best wishes for the authors!: | Thank you very much for your insightful comments and feedback. We believe that it helps to enhance the quality of the manuscript. We add the forest plot. | The revised texts are in “red”. Page: 9 |

In conclusion, the revised version of the manuscript has been produced as per the review outcomes. So, we hope that you will be happy to see this greatly improved version. Once again, we would like to thank you all for your dedication, professional services, and cooperation.
